# Bronchial asthma and chronic obstructive pulmonary disease: research activity in Arab countries

**DOI:** 10.1186/2049-6958-9-38

**Published:** 2014-07-08

**Authors:** Waleed M Sweileh, Samah W Al-Jabi, Sa’ed H Zyoud, Ansam F Sawalha

**Affiliations:** 1 Department of Pharmacology/Toxicology, College of Medicine and Health Sciences, An-Najah National University, Nablus, Palestine; 2 Department of Clinical and Community Pharmacy, College of Medicine and Health Sciences, An-Najah National University, Nablus, Palestine

**Keywords:** Arab countries, Bronchial asthma, COPD, Scopus

## Abstract

**Background:**

Chronic respiratory diseases, like bronchial asthma and chronic obstructive pulmonary disease (COPD), are a worldwide health problem. Quantitative and qualitative assessment of asthma and COPD-related research from Arab countries has not been explored and there are few internationally published reports on such field. The main objectives of this study were to analyze research output originating from Arab countries in the field of bronchial asthma and COPD.

**Methods:**

Original scientific articles or reviews published from the 22 Arab countries were screened using specific words pertaining to asthma and COPD using Scopus database and search engine. Research productivity was evaluated based on: (a) total and trends of contribution of each Arab country to asthma and COPD research and (b) journals in which researchers from Arab countries published their research.

**Results:**

The total number of original research and review articles published globally about bronchial asthma and COPD was 163,964. The leading country in bronchial asthma and COPD research was United States of America (38,632; 23.56%). Worldwide, Turkey ranked 19th while Israel and Iran ranked 25th and 29th respectively. Among Arab countries, Egypt and Kingdom of Saudi Arabia came on positions 39th and 43rd, respectively. A total of 1,304 documents about bronchial asthma and COPD were published from Arab countries which represents 0.8% of the global research output. Research in bronchial asthma was almost double that in COPD. Research from Arab countries was low and showed a significant increase after 2000. Approximately 12% of research activity in asthma and COPD from Arab countries was published in *Saudi Medical Journal, Annals of Saudi Medicine, Eastern Mediterranean Health Journal* and *Tunisie Medicale*. Kingdom of Saudi Arabia, with a total publication of 353 (27.07%) ranked first among the Arab countries while University of Kuwait was the most productive institution with a total of 123 (9.43%) documents.

**Conclusions:**

The present data showed relatively low research productivity about bronchial asthma and COPD in Arab countries. Research output can be improved by investing more in international and national collaborative research projects in the field of asthma and COPD.

## Background

Chronic respiratory diseases are chronic diseases of the airways and other parts of the lung. Bronchial asthma and chronic obstructive pulmonary disease (COPD) are the most common types of chronic respiratory diseases [1]. The global burden of asthma and COPD is increasing and it is estimated that more than 500 million people suffer from bronchial asthma and COPD (http://www.who.int/respiratory/en/) [2]. The world health organization (WHO) is leading a global effort to expand understanding of chronic obstructive pulmonary disease (COPD) and advocate for better patients care. One such aspect of fighting COPD is carried out through the “Global Alliance against Chronic Respiratory Diseases” (GARD) which is a global alliance dedicated to reduce the global burden of COPD (http://www.who.int/respiratory/gard/en/). The WHO also plays a major role in coordinating international efforts against bronchial asthma to reduce the disability and premature death related to asthma particularly in low and middle income member countries. Another global alliance for asthma is GINA which was launched in 1993 in collaboration with the National Heart, Lung, and Blood Institute, National Institutes of Health, USA, and the World Health Organization (http://www.ginasthma.org/).

Research about bronchial asthma and COPD is one important aspect that each country needs to provide in the struggle against such emotionally difficult type of chronic diseases. Research in chronic respiratory diseases will shed light on the environmental and genetic risk factors as well as cultural and regional differences in the epidemiology of such diseases. An important goal of medical research is to help in initiating communication, exchange of findings and building a network of scientists sharing similar research interest. One thoughtful approach to achieve and implement such goal is through assessing and evaluating research productivity in a particular field or particular disease. It is believed that the quality, quantity and type of research publications shape up the prestige of the researcher and the research institution. Furthermore, the annual number and type of research from a particular institute or country reflects the health concern and health agenda of that country. Research about bronchial asthma and COPD can positively reflect on clinical practice in any particular region.

One method to assess research contribution from any country is bibliometric analysis which refers to the implementation of statistical methods for evaluating the research productivity, for individuals, institutes and countries [3]. Bibliometric analysis is a useful tool to obtain information about the current state of research in particular areas [4-6]. Bibliometrics has been applied to various diseases and is now widely accepted as a method of measuring research and literacy output in any particular area [7-14]. Therefore, the objective of this study was to analyze research output from 22 Arab countries about bronchial asthma and COPD. The Arab countries cover a large geographic area including North Africa and the Arabic Peninsula with around 380 million inhabitants. Up to the authors’ knowledge, no bibliometric studies have been published from the Arab world about bronchial asthma and/or COPD. Such study will lead to a better understanding of the past, current and future status of research in chronic respiratory diseases in the Arab world which, hopefully, can lead to better preventive disease strategies and better patient-oriented health services [15-19]. The results of the study will help health policy makers and people in academia and clinical practice to shape up bronchial asthma and COPD research in the future. In addition, research activity needs to be encouraged and maintained through analysis of publications from researchers so as to provide feedback to health policy makers and education planners.

## Methods

### Search strategy

The data used in this study were based on the Scopus online database. A comprehensive online search was performed using SciVerse Scopus, which is one of the world’s largest databases of peer-reviewed literature. Scopus covers nearly 18,000 titles from 5,000 publishers worldwide, contains 41 million records and provides 100% coverage of MEDLINE [20]. Elsevier, combining the characteristics of both the Web of Science and PubMed, developed the Scopus database. These characteristics allow for an enhanced service for educational and academic needs, as well as for medical literature research and bibliometric analysis. Scopus offers basic search and advanced search features. In the basic search, the results for the chosen keywords can be limited by the date of publication, subject area or document type [12,21]. The search output from Scopus can be presented as a list of 20–200 items per page and extracted documents can be exported to Microsoft Excel®. The results can be refined by document type, author name, source title and publications per year and/or subject area. In addition, a new search can be initiated within the results [21,22].

The keywords entered into the Scopus search engine to achieve the objectives of this study were entered as part of the “Article Title” or “Abstract”. All subject areas were selected for this search: health sciences, social sciences, life sciences and physical sciences, including all previous years up to the date of December 31st, 2012. The resulting search was as follows: AFFILCOUNTRY(qatar) OR AFFILCOUNTRY(jordan) OR AFFILCOUNTRY(egypt) OR AFFILCOUNTRY(emirates) OR AFFILCOUNTRY(saudi) OR AFFILCOUNTRY(palestine) OR AFFILCOUNTRY(bahrain) OR AFFILCOUNTRY(yemen) OR AFFILCOUNTRY(syrian) OR AFFILCOUNTRY(iraq) OR AFFILCOUNTRY(kuwait) OR AFFILCOUNTRY(oman) OR AFFILCOUNTRY(lebanon) OR AFFILCOUNTRY(morocco) OR AFFILCOUNTRY(tunisia) OR AFFILCOUNTRY(sudan) OR AFFILCOUNTRY(algeria) OR AFFILCOUNTRY(comoros) OR AFFILCOUNTRY(djibouti) OR AFFILCOUNTRY(libya) OR AFFILCOUNTRY(mauritania) OR AFFILCOUNTRY(somalia) AND (TITLE(asthma) OR ABS(asthma) OR TITLE(asthmatic) OR ABS(asthmatic) OR TITLE(chronic obstructive pulmonary disease) OR ABS(chronic obstructive pulmonary disease) OR TITLE(chronic obstructive lung disease) OR ABS(chronic obstructive lung disease) OR TITLE(copd) OR ABS(copd) OR TITLE(emphysema) OR ABS(emphysema) OR TITLE(chronic bronchitis) OR ABS(chronic bronchitis)).

We excluded documents that were published as an erratum. Scientific research productivity in the years 2013 and 2014 was excluded because these years were still open for new journal issues and therefore inclusion of the year 2013 would create error and bias. All searches were completed on March 28, 2014 to avoid bias due to the daily updating of the database.

Scientific output was evaluated based on a methodology developed and used in other bibliometric studies [12,23-26]. The collected data were used to generate the following information: (a) total and trends of contributions in asthma and COPD research during all previous years up to December 31st, 2012; (b) research productivity by country; (c) journals in which Arab researchers published; (d) the productivity and impact of the most prolific institutions; and (e) the citations received by the publications.

### Indices of research productivity

The measurements of bibliometric analysis (e.g. countries, cited articles, institutions) were converted to the rank order using the standard competition ranking (SCR). We took in our consideration only the ten top-ranked. If the measurements of bibliometric analysis have the same ranking number, then a gap is left in the following ranking numbers. The *h*-index for data collected from *Scopus* is presented. The *h*-index is a country’s number of articles (h) that have received at least h citations. It quantifies both country scientific productivity and scientific impact and it is also applicable to scientists, journals, etc. [27]. That is to say, a country with an h-index of 10 has published 10 documents, each have attracted at least 10 citations. Documents with fewer than 10 citations are not calculated by the index. The *h*-index was originally developed as a way of qualifying research performance [28]. Journal impact factor (IF) was evaluated using the Journal Citation Report (JCR; Web of Knowledge) 2012 science edition by Thomson Reuters (New York, NY, USA).

### Ethical approval

The Institutional Review Board (IRB) at An-Najah National University does not require submission of an IRB application for a bibliometric study. The IRB confirmed that there is no risk to human subjects in this type of research since the data are based on published literature and, as secondary data, did not involve any interactions with human subjects.

### Statistical analysis

Data from Scopus were exported to Microsoft Excel® and then transferred to the Statistical Package for Social Sciences, Version 15 (SPSS; SPSS Inc., Chicago, IL, USA) program for analysis.

## Results

**Figure 1:**
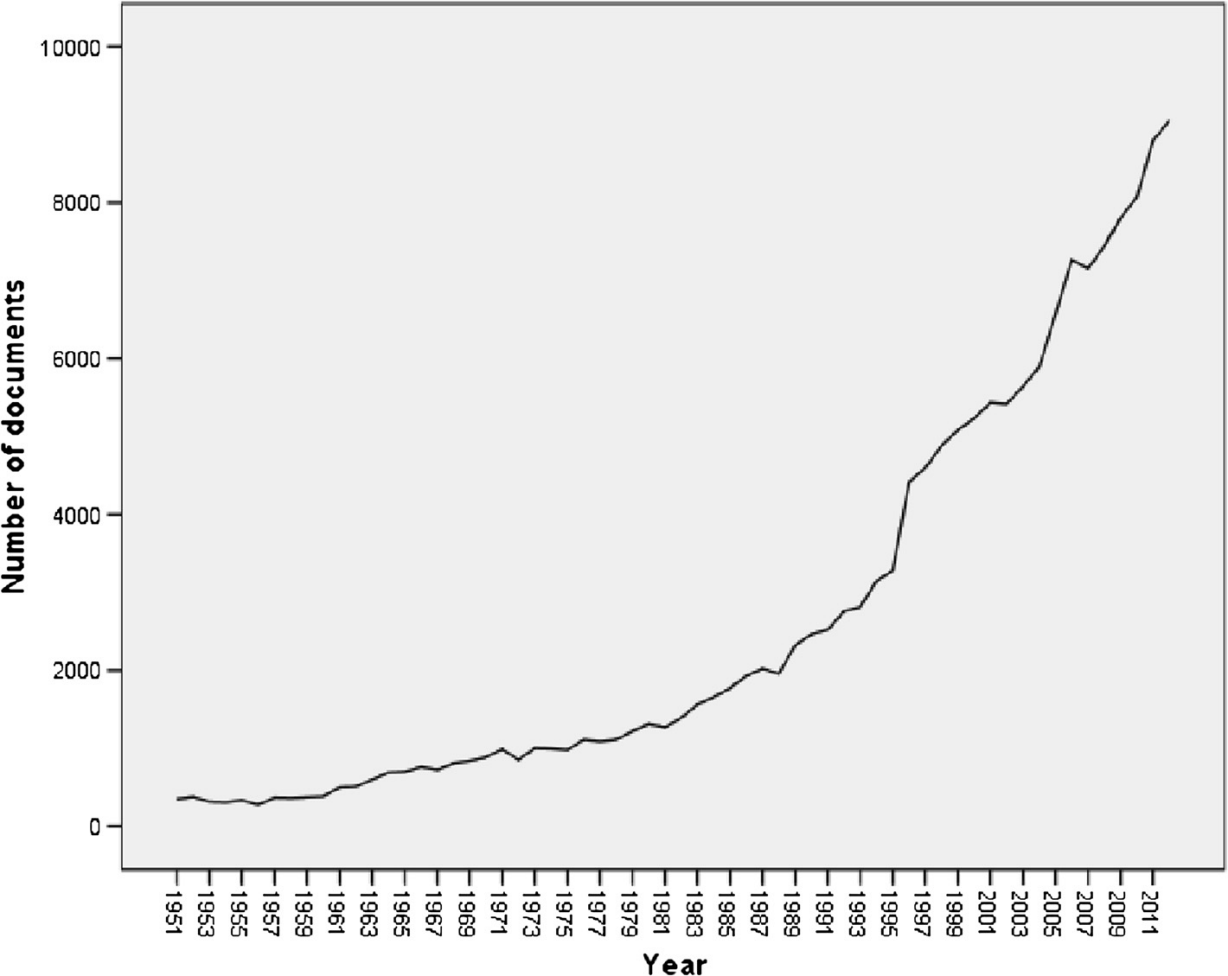
Worldwide research productivity in bronchial asthma and COPD as extracted from Scopus database.

**Figure 2:**
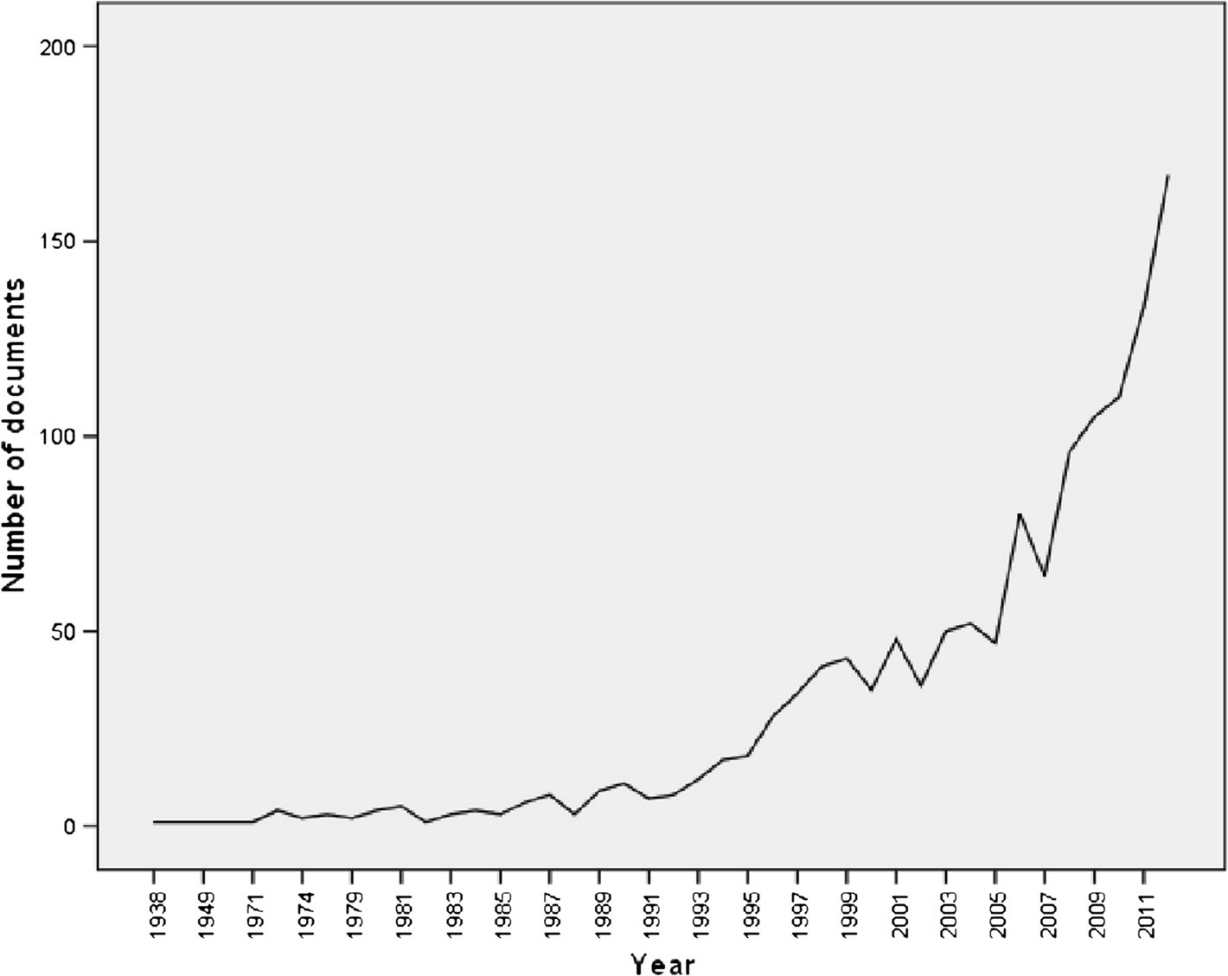
Research productivity about asthma and COPD from Arab countries as extracted from ISI Web of Science.

The total number of original and review documents retrieved from Scopus without specifying the name of any country and using the keywords listed in the methodology was 163,964. This number represents the global research productivity (original research articles and reviews) in bronchial asthma and COPD using the key words listed in methodology. The leading countries in bronchial asthma and COPD research were United States of America (USA) (38,632; 23.56%) followed by United Kingdom (UK) (15,535; 9.47%) and Japan (8,092; 4.93%). Worldwide, Turkey ranked 19th while Israel and Iran ranked 25th and 29th respectively. Among Arab countries, Egypt and Kingdom of Saudi Arabia (KSA) ranked 39th and 43rd respectively. The 163,964 documents were written in 41 different languages, mainly English (126,408; 77.09%) followed by German, French and Japanese languages with a total of 6,428 (3.92%); 5,025 (3.06%); and 4,763 (2.90%) respectively. The annual global research productivity about bronchial asthma and COPD showed steady growth over the past 50 years. The number of documents published in 2005 was approximately 7 folds more than that published in 1975 (Figure 1). The majority of globally published documents were in the subject area of Medicine (137,825; 84.05%) followed by Immunology and Microbiology (17,671; 10.78%); Biochemistry, Genetics and Molecular Biology (13,312; 8.12%) and Pharmacology, Toxicology and Pharmaceutics (11,747; 7.16%). The most common journals in which asthma and COPD documents appeared were *Journal of Allergy and Clinical Immunology*; *Chest*; and *Thorax* with total number of documents of 5,537 (3.37%), 4,167 (2.54%), and 3,144 (1.92%) respectively. The most productive institutions of asthma and COPD research were VA Medical Center (USA), National Heart and Lung Institute (USA), and Brigham and Women’s Hospital with a total of 1,904 (1.16%), 1,481 (0.90%), and 1,299 (0.79%) documents respectively.When the same methodology was applied using the list of the 22 Arab countries, 1,304 asthma and COPD documents were retrieved. Therefore, research about asthma and COPD published from Arab countries represents approximately 0.80% of the global research productivity in the field. Out of the 1,304 documents, 1,152 (88.34%) were written in English language. The annual number of documents published from Arab countries indicated that research activity in this field was low and steady until early 2000 and showed a sharp increase in the last decade (Figure 2).

When retrieved data were analyzed by country, Kingdom of Saudi Arabia (KSA) (353; 27.07%) had the highest research output followed by Egypt (242; 18.56%) and Tunisia (169; 12.96%). More than half (58.58%) of asthma and COPD research from Arab world came from the three Arab countries; KSA, Egypt and Tunisia. No data related to asthma and COPD were found from Somalia, Djibouti, Mauritania and Comoros (Table 1). The first article about asthma and/or COPD published from an Arab country appeared in 1938 (Bilharzial asthma. Bronchial asthma in Schistosoma infection) [29]. Collaboration between Arab countries and non-Arab countries in asthma and COPD research and publication was evident. Countries whose researchers collaborated most with investigators in the Arab world include France (89; 6.8%), United States of America (USA) (87; 6.67%), and England (81; 6.21%).

**Table 1:** Contribution of each Arab country to the 1,304 published documents about asthma and COPD

**Country**	N (%)*
Saudi Arabia	353 (27.07)
Egypt	242 (18.56)
Tunisia	169 (12.96)
Kuwait	130 (9.97)
United Arab Emirates	99 (7.59)
Morocco	92 (7.05)
Lebanon	76 (5.83)
Jordan	54 (4.14)
Qatar	46 (3.52)
Algeria	40 (3.06)
Iraq	38 (2.91)
Oman	28 (2.14)
Syrian Arab Republic	27 (2.07)
Sudan	23 (1.76)
Bahrain	12 (0.9)
Palestine	11 (0.8)
Yemen	3 (0.2)
Libyan Arab Jamahiriya	2 (0.15)
Somalia	0
Comoros	0
Djibouti	0
Mauritania	0

*Total exceeds 100% because of overlap in some documents among more than one Arab country.

The majority of published documents from Arab countries were in the subject area of Medicine (1,095; 83.97%). Top 10 subject areas of research interest of asthma and COPD publications from Arab countries are shown in Table 2. The key word “asthma” was used in 806 documents while COPD key word was used in 355 documents. The 1,304 documents were published in 160 different journals. Table 3 lists the top 10 journals in which documents about asthma and COPD from Arab countries were published. One hundred and fifty eight (12.11%) documents appeared in 4 medical journals that are based in Arab countries, particularly *Saudi Medical Journal, Annals of Saudi Medicine, Eastern Mediterranean Health Journal* and *Tunisie Medicale.* The 4 journals have a wide medical scope. Out of the top 10 journals, 3 (*Annals of Thoracic Medicine; Respiratory Medicine; Revue Des Maladies Respiratoires*) were in the specific field of Respiratory/ Thoracic medicine whereas 2 were in allergy/ immunology and 1 in the field of asthma (*Journal of Asthma*). Interest of Arab gulf researchers in asthma and COPD research is evident in top 10 Arabic institutions involved in asthma and COPD research (Table 4). The most productive institution was University of Kuwait (123; 9.43%) followed by King Saud University in KSA and King Faisal Specialist Hospital and Research Centre KSA with a total of 114 (8.74%) and 65 (4.98%) publications respectively. Except for the King Faisal Specialist Hospital Research Centre, all institutions in the top 10 list were academic institutions. Five of the top 10 institutions were based in the Arab Gulf and 3 were based in Egypt while the remaining 2 were based in Jordan and Lebanon. The total number of citations for bronchial asthma and COPD documents from the Arab world, at the time of data analysis (April 12th, 2014), was 13,354. Out of the 1304 documents considered for the *h*-index, 44 had been cited at least 44 times at the time of data analysis.

**Table 2:** Research areas of the 1,304 documents about asthma and COPD published from the 22 Arab countries

**SCR**	**Research area**	**Number (%)**
		**N = 1,304**
**1st**	Medicine	1,095 (83.97)
**2nd**	Immunology and Microbiology	121 (9.27)
**3rd**	Pharmacology, Toxicology and Pharmaceutics	120 (9.2)
**4th**	Biochemistry, Genetics and Molecular Biology	101 (7.74)
**5th**	Agricultural and Biological Sciences	40 (3.06)
**6th**	Environmental Science	33 (2.5)
**7th**	Nursing	22 (1.68)
**8th**	Veterinary	16 (1.22)
**9th**	Neuroscience	16 (1.22)
**10th**	Chemistry	14 (1.07)

*SCR* Standard Competition Ranking.

**Table 3:** Top 10 journals in which documents about asthma and COPD were published from the 21 Arab countries

**SCR**^**a**^	**Journal**	**Number (%)**	IF^*^
		**N = 1,304**	
**1st**	*Saudi Medical Journal*	62 (4.7)	0.619
**2nd**	*Annals of Saudi Medicine*	36 (2.7)	1.103
**2nd**	*Journal of Asthma*	34 (2.6)	1.848
**4th**	*Tunisie Medicale*	33 (2.5)	NA
**5th**	*Revue Francaise D Allergologie Et D Immunologie Clinique*	29 (2.2)	NA
**6th**	*Annals of Thoracic Medicine*	28 (2.1)	1.123
**7th**	*Eastern Mediterranean Health Journal*	27 (2.1)	NA
**8th**	*Respiratory Medicine*	21 (1.6)	2.585
**8th**	*Journal of Allergy and Clinical Immunology*	20 (1.5)	12.047
**8th**	*Revue Des Maladies Respiratoires*	17 (1.3)	0.495

*SCR* Standard Competition Ranking, *IF* impact factor.

^*^The impact factor was reported according to Institute for Scientific Information (ISI) journal citation reports (JCR) 2012.

^a^Equal journals have the same ranking number, and then a gap is left in the ranking numbers.

**Table 4:** Top 10 active institutions in hypertension research from Arab countries

**SCR**^**a**^	**Institution**	**Number (%)**	**Affiliation**
		**N = 1,304 (100%)**	
**1st**	University of Kuwait	123 (9.4)	Kuwait
**2nd**	King Saud University	114 (8.7)	KSA
**3rd**	King Faisal Specialist Hospital and Research Centre	65 (5.0)	KSA
**4th**	Cairo University	54 (4.1)	Egypt
**5th**	United Arab Emirates University	53 (4.1)	UAE
**6th**	Ain Shams University	51 (3.9)	Egypt
**7th**	Centre Hospitalier Universitaire Ibn-Rochd	36 (2.8)	Morocco
**8th**	Hopital de Pneumo-Phtisiologie Abderrahman-Mami	35 (2.7)	Tunisia
**9th**	King Abdulaziz University	33 (2.5)	KSA
**10th**	CHU Fattouma-Bourguiba	30 (2.3)	Tunisia

*KSA* Kingdom of Saudi Arabia, *SCR* Standard Competition Ranking, *UAE* United Arab Emirates.

## Discussion

Chronic respiratory diseases are considered a worldwide health problem. The prevalence of chronic respiratory diseases in Arab countries is believed to be increasing in the light of urbanization and industrialization especially in certain rich Arab countries. With more funds and governmental financial support to medical research, more accurate publications about epidemiology and triggering factors are expected to appear with regard to chronic respiratory diseases in Arab countries. Recent literature indicated that non-communicable diseases, including COPD, are becoming the real future health challenge and burden for Arab countries [30]. Several epidemiological studies indicated that bronchial asthma is common in Arab world and is mostly associated with allergic disorders [31-59]. Furthermore, bronchial asthma is affecting both children and adults and is imposing an economic burden on individuals and on health systems [60]. In contrast to asthma, epidemiological studies about COPD in Arab countries are fewer and most information regarding COPD in Arab countries is obtained from BREATHE study which indicated that the prevalence of COPD was lower than that reported in developed countries mostly due to under-reporting or the presence of other non-smoking factors [61-66]. Screening the net showed that there is one poster published in a scientific conference (Dhubai, UAE in 2010) regarding research activity about asthma in Arab world. The authors of the poster used PubMed to screen for asthma publications and found that the number of asthma related publications originating from Arab countries over the last 10 years totaled 275 articles (https://wao.confex.com/wao/2010isc/webprogram/Paper1414.html). However, up to the author’s best knowledge, there are no published full articles about bibliometric analysis of research activity regarding respiratory system, respiratory medicine, asthma and/ or COPD in Arab countries. However, several articles were published from other parts of the world [67-72]. These international publications are important for readers in the field of respiratory medicine, public health and health system research.

Reducing chronic respiratory diseases-related morbidity and mortality in Arab countries requires periodic assessment of how these various countries progress in scientific research pertaining to epidemiology, awareness, control and risk factors associated with asthma and COPD. Furthermore, identifying research output and research activity in asthma and COPD is of great importance to public health and pharmaceutical industry. Better delivery systems might help increase compliance and therapeutic outcomes among patients with asthma/ COPD. The main goal of such a study is to direct attention of professionals, academics, researchers and pharmaceutical industry to the current status of asthma and COPD research activity. Academic institutions in the Arab world are advised to initiate more research about bronchial asthma and COPD and to strengthen research collaboration with international researchers and institutions. For future studies in this direction, it is recommended that similar quantitative and qualitative research analyses for other disciplines, particularly allergy and immunology is needed.

Our study was based on 1,304 documents extracted from Scopus, and therefore, cannot be generalized to bronchial asthma and COPD literature published from other Arab countries covered by other databases such as Google Scholar. However, the study does give a clear and a close picture about the characteristics of published asthma and COPD documents from Arab countries. Our results indicated that asthma research is almost double that of the COPD and more efforts are needed to promote research about COPD. Our results also showed that the majority of top 10 institutions in asthma and COPD research were universities with medical schools. Research productivity reveals intellectual output by the institution and is useful to university administrators when evaluating performance of university faculties in the light of university ranking among various universities [73].

Our study is not without limitations, most of which are the same as those of studies performed in other biomedical fields [11,26,74,75]. First of all, articles published in journals not indexed in Scopus were not included in the analysis, although they might contribute to scientific productivity. Another limitation is the keywords used to screen for asthma and COPD publications. The authors did their best to include all possible and commonly used terms in this field. However, some articles might have been missed because they might have used some keywords that are different than the ones used in our research methodology. Finally, it should be noted that research output for certain institutions could have been under-estimated because of writing their English names differently in different articles. Therefore, such authors might have 2 or more institute profiles in Scopus database because their names were written differently in different articles.

## Conclusion

The present data showed that Arab countries have relatively low research productivity in the field of asthma and COPD. Research output can be improved by investing in more international and national collaborative research projects. Pharmaceutical industry should benefit from the data presented and invest in research leading to the transfer of recent technology pertaining to asthma and COPD therapy.

## Abbreviations

SPSS: Statistical Package for Social Sciences; ISI: Institute for Scientific Information; KSA: Kingdom of Saudi Arabia; UAE: United Arab Emirates; SAR: Syrian Arab Republic; USA: United States of America; WHO: World Health Organization; JCR: Journal Citation Report; IRB: Institutional Review Board; SCR: Standard Competition Ranking; IFs: Impact factors.

## Competing interests

The authors declare that they have no competing interests.

## Authors’ contributions

All authors were involved in drafting the article, and all authors approved the final version to be submitted for publication. WS and SZ contributed to idea and design while S.A and A.S contributed to data analysis, critical thinking and manuscript writing and submission.
